# The secondary loss of gyrencephaly as an example of evolutionary phenotypical reversal

**DOI:** 10.3389/fnana.2013.00016

**Published:** 2013-06-26

**Authors:** Iva Kelava, Eric Lewitus, Wieland B. Huttner

**Affiliations:** Max Planck Institute of Molecular Cell Biology and GeneticsDresden, Germany

**Keywords:** brain evolution, neocortex, lissencephaly, gyrencephaly, reverse evolution

## Abstract

Gyrencephaly (the folding of the surface of the neocortex) is a mammalian-specific trait present in almost all mammalian orders. Despite the widespread appearance of the trait, little is known about the mechanism of its genesis or its adaptive significance. Still, most of the hypotheses proposed concentrated on the pattern of connectivity of mature neurons as main components of gyri formation. Recent work on embryonic neurogenesis in several species of mammals revealed different progenitor and stem cells and their neurogenic potential as having important roles in the process of gyrification. Studies in the field of comparative neurogenesis revealed that gyrencephaly is an evolutionarily labile trait, and that some species underwent a secondary loss of a convoluted brain surface and thus reverted to a more ancient form, a less folded brain surface (lissencephaly). This phenotypic reversion provides an excellent system for understanding the phenomenon of secondary loss. In this review, we will outline the theory behind secondary loss and, as specific examples, present species that have undergone this transition with respect to neocortical folding. We will also discuss different possible pathways for obtaining (or losing) gyri. Finally, we will explore the potential adaptive consequence of gyrencephaly relative to lissencephaly and vice versa.

## Introduction

The mammalian brain, and especially its evolutionarily newest part, the neocortex, has intrigued biologists for centuries. The complexity of this organ gave rise to numerous research fields, and our efforts to understand its building blocks and the synergy with which they operate has resulted in a vast amount of knowledge on most of the brain's biology. Numerous researchers and their work have shed light on neurogenesis, neuronal connectivity, memory formation and storage and processing. Yet, far less effort has been devoted to elucidating the formation of the brain's morphology, despite interspecific diversity in this regard. Furthermore, the outer morphology of the mammalian brain is unique among the vertebrates, as it is only mammals who exhibit such variety in the appearance of their brain.

**Figure 1:**
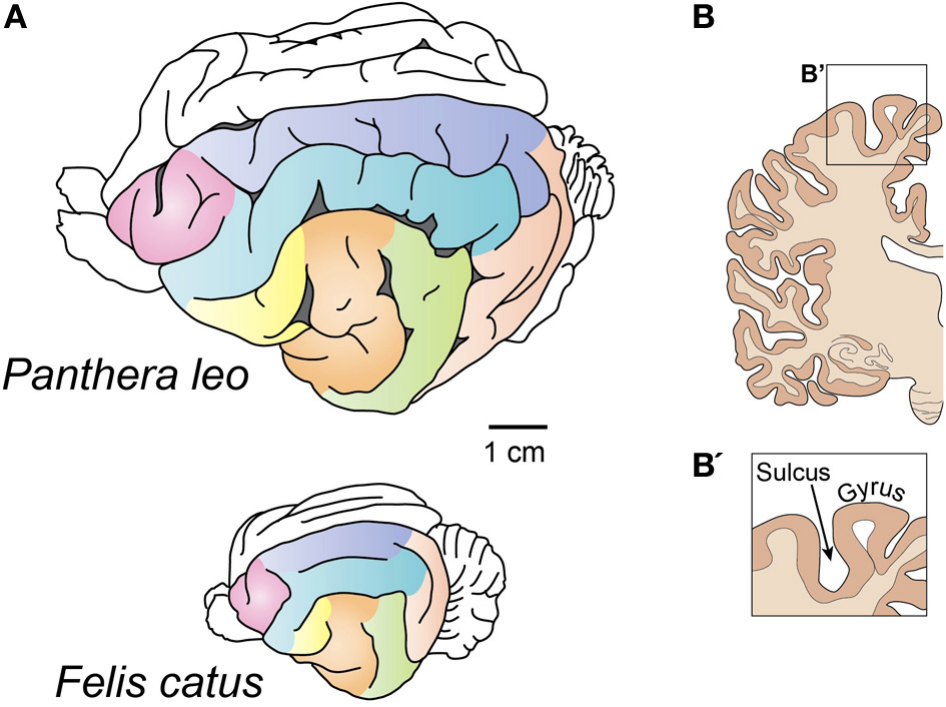
**Gyrencephalic brains. (A)** Brains of the lion (top) and the house cat (bottom). Despite a different level of gyrification, note the similarities in the pattern of gyri and sulci distribution. After Welker (1990). Homologous gyri are colored to facilitate comparison. **(B)** A coronal section of the human brain showing gryi and sulci. This image does not follow the scale bar in **(A)**.

The neocortex can be either smooth (*lissencephalic*) or folded into numerous convolutions (*gyrencephalic*) (Figure 1A). These convolutions are called *gyri* (Sg. *gyrus*), and the spaces between them are termed *sulci* (Sg. *sulcus*) (Figure 1B). Several hypotheses as to why the surface of the neocortex folds have been proposed (Zilles et al., 2013), but none of these proposals are fully able to explain the mechanism of its genesis or to shed light on the adaptive value of having or not having a folded neocortex (see section Why Does the Neocortex Fold?).

The distribution of gyri and sulci is conserved among members of the same order of mammals (Figure 1A) (Welker, 1990), and it is only the smaller gyri that show individual variation. This remarkable feature of the brain has been implicated in cognitive abilities (Jerison, 1973), but how these two traits connected and whether this relationship is universal has not been shown. Gyrencephaly has also been shown to correlate with brain weight and cortical thickness (Pillay and Manger, 2007; Manger et al., 2012), but several outliers exist that show that this correlation is not exclusive and speak in favor of an adaptive nature of brain folding.

## Why does the neocortex fold?

If one considers Cope's rule (Cope, 1896), the reason why the neocortex folds is quite obvious. Lineages which follow this rule increase in body size during evolution, and therefore their brains must also increase in size. Because of the biomechanical constraint (the stress imposed on the cervical vertebrae with the increasing size of the head), the head cannot scale isometrically with the body, but becomes relatively smaller. Likewise, the thickness of the neocortex cannot increase, due to a constraint on the pattern of connectivity; yet an enlarging body requires a larger number of neurons. The problem is solved by expanding the surface or the neocortex, not its thickness. This phenomenon is called *ballooning*. An expanded neocortex is therefore fit into a smaller skull by way of folding (Striedter, 2005).

A simple explanation of the gyrification would therefore be that it is the spatial constraint of the skull that forms the fold in the neocortex. That this is the only reason for folding was refuted more than 50 years ago, following experiments in which a part of the skull was removed from the head of the sheep embryo (Barron, 1950). Despite the invasive procedure, the folds on the sheep's brain developed without much disturbance. As we mentioned before, the pattern of gyri distribution is remarkably constant, which speaks against the simple explanation of the skull being an obstruction to the neocortex's expansion.

The fact that in extant species gyrification is not driven by the constraint of the skull does not mean that the trait itself did not appear because of spatial limitations. In the earliest mammals, the ballooning of the neocortex could have been solved by folding it, and the genetic program involved in the folding might have been fixed in subsequent lineages by genetic assimilation (Waddington, 1961).

**Figure 2:**
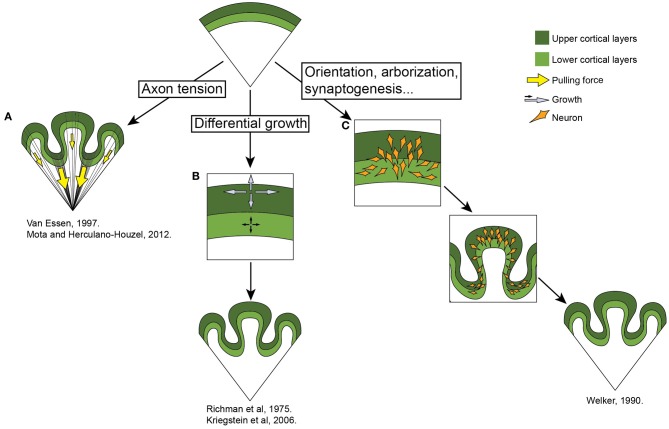
**Theories of gyrogenesis. (A)** Neuronal tension (Van Essen, 1997; Mota and Herculano-Houzel, 2012). This theory stresses the differential pulling forces of the underlying white matter on the neocortex, resulting in the convoluted appearance of the brain. **(B)** Differential growth (Richman et al., 1975; Kriegstein et al., 2006) theory states that the folding of the neocortex is a result of the differential expansion of uppers versus lower neuronal layers. **(C)** Welker (1990) emphasizes various processes involved in gryogenesis, including the orientation of neurons, their arborization and incoming fibers. See main text.

The exact reason for the development of folded neocortices, especially in an evolutionary context, is still elusive. But several attempts have been made to understand the mechanical properties of its genesis. At the moment there are several theories regarding the ontogeny of gyri. Some researchers stress the importance of the white matter, which underlies the cortex and plays a crucial role in the folding of the neocortex (Van Essen, 1997; Mota and Herculano-Houzel, 2012). This mechanism is based on the pulling force that the axonal fibers exert on the overlying neocortex. An opposing view was taken by Richman (Richman et al., 1975; Kriegstein et al., 2006) who placed emphasis on the neocortical gray matter itself. In this theory, differential growth rates between the superficial and the deeper layers of neurons are what cause the developing neocortex to fold. Welker (1990) proposes that the gyral and sulcal areas of the brain differ in their architectural complexity. According to this theory, several processes contribute to the formation of gyri in specific, predetermined places. These include, among others, dendrogenesis, neuronal orientation, afferent arrival, arborization, synaptogenesis. The present theories are summarized in Figure 2. As the process of gyrogenesis is very difficult to examine *in vivo*, most of the approaches are theoretical or purely descriptive (Smart and McSherry, 1986a,b) and none of them has fully succeeded in explaining the mechanism itself, nor the precise determination of the pattern. The ontogenesis of gyri is probably a very complex process which includes the predetermination of gyri position early in development by patterning the early neural tube and influencing the distribution of progenitor cells, followed by a combination of mechanical processes spanning all gyrogenesis hypotheses.

The functional significance of a gyrified brain is also somewhat unclear. It has been noted that the pattern of gyri sometimes corresponds to the borders of different cortical areas (Welker, 1990, and references within). The firmly set pattern of gyri and sulci distribution speaks in favor of them being indispensable for normal establishment of neuronal connections and cortical areas [although, individuals with severe disruptions of gyrification pattern survive (Olson and Walsh, 2002; Singer et al., 2013)].

## Can the neocortex “unfold”?

The classical view of neocortex evolution states that the evolution of the mammalian brain followed a linear path (a sort of *scala naturae*), emerging from a small, lissencephalic brain of the earliest mammals, and evolving into a large, highly folded brain (Striedter, 2005; Rakic, 2009). This view has been disputed by recent paleontological and molecular analyses which state that the ancestor of mammals was, in fact, relatively large-bodied (Luo, 2007; Romiguier et al., 2013). The simplistic trajectory of brain evolution was further questioned by the dissection of the cell-biological properties of progenitors giving rise to neurons. With a different picture of the ancestral brain, additional courses of brain evolution came to light. Moreover, the view that the brain evolved simply from a lissencephalic one to a gyrencephalic one would imply that gyrencephaly evolved independently in all mammalian orders. The more parsimonious explanation for the vast differences in the morphology of the neocortex is that the ancestor of the mammals was gyrencephalic, and that some lineages underwent a type of a phenotypic reversal, becoming secondarily lissencephalic (Kelava et al., 2012).

In all mammalian species, most neocortical neurons are born during embryonic development (in some species this process extends into an early postnatal period). Neurons are born from several progenitor types which possess different cell-biological properties and, hence, different proliferative and neurogenerative capacities [for reviews on mammalian neural progenitors, see (Götz and Huttner, 2005; Lui et al., 2011; Kelava and Huttner, 2012)]. The subventricular zone (SVZ) of the embryonic neocortex produces most of the neocortical neurons (Haubensak et al., 2004; Miyata et al., 2004; Noctor et al., 2004, 2008). This zone varies greatly in size and neural progenitor composition among different species. It is thought that it is these differences in the architecture of the SVZ have enabled the increase in size of the neocortex in some lineages (Kriegstein et al., 2006). Most of the efforts in elucidating the brain's potential for growth have been concentrated on studying the brain of primates, with special interest in the human brain (Rakic, 1988; Smart et al., 2002; Fietz et al., 2010; Hansen et al., 2010). These efforts led to the discovery of a novel progenitor type in the human developing brain (Fietz et al., 2010; Hansen et al., 2010)—the basal radial glia (bRG) (also called outer radial glia and intermediate radial glia). Further developments have described this population in the ferret (Fietz et al., 2010; Reillo et al., 2011), mouse (Shitamukai et al., 2011; Wang et al., 2011), rat (Martínez-Cerdeño et al., 2012), marmoset (García-Moreno et al., 2012; Kelava et al., 2012), and the macaque (Smart et al., 2002; Martínez-Cerdeño et al., 2012). This progenitor population, after being found in gyrencephalic species at a higher abundance, was thought to be required for the expansion of the neocortex observed in the gyrencephalic brains. The discovery of these cells in a lissencephalic species, the marmoset, at a similar level to gyrencephalic species, inspired us to consider additional explanations. One of which was secondary lissencephaly. An investigation into the occurrence of lissencephalic versus gyrencephalic species across the mammalian phylogeny brought about several lineages which could also potentially be secondarily lissencephalic.

## Examples of secondary lissencephaly

Since lissencephaly was previously thought to be a primitive trait, species with smooth neocortices were concordantly also portrayed as primitive. Recent advances in the field of brain evolution show that, for at least some of the species, the lissencephalic neocortex might actually be a derived trait.

**Figure 3:**
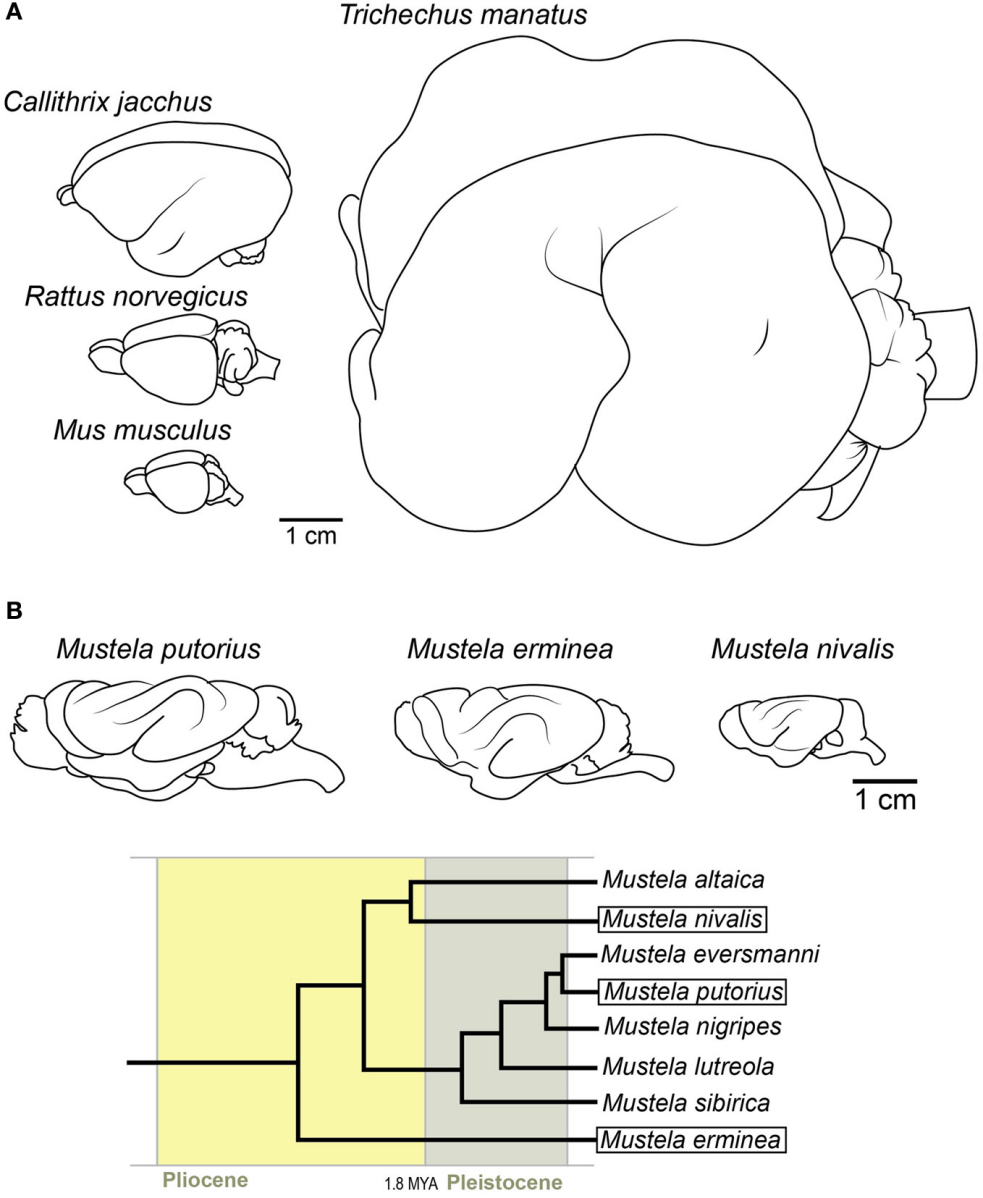
**Examples of potentially secondarily lissencephalic brains. (A)** Top left: Common marmoset, middle left: rat, bottom left: mouse. Right: West Indian manatee. **(B)** Brains of three extant mustelid species. Note the simplification of the gyrification pattern in smaller brains. The gyri in smaller brains are also shallower. Below is the phylogenetic tree [after Koepfli et al. (2008)] showing the relationships between these species and their time of divergence.

The marmoset might be the best described example of an animal which underwent a phenotypic reversal with regard to brain morphology (Kelava et al., 2012) (Figure 3A). The developing marmoset neocortex was shown to have a very similar cytoarchitecture to a developing gyrencephalic neocortex, thus contrasting the then present view of a high abundance of bRG being gyrencephaly-specific (García-Moreno et al., 2012; Kelava et al., 2012). This established that a high abundance of bRG does not necessarily correlate with gyrencephaly. The marmoset's unusual development and its physiology inspired us to investigate other possible ways in which these two traits were connected. The marmoset family (Callithricidae) belongs to New World monkeys (Platyrrhini) and is characterized by its small body size. It has been proposed that this small body size is actually not a primitive trait, but that the whole lineage evolved by phyletic dwarfing (Ford, 1980; Montgomery and Mundy, 2013). The notion that the marmoset evolved from a big-bodied, gyrencephalic ancestor finds support in ancestral reconstructions based on these traits in monkeys and apes (Kelava et al., 2012) and, therefore, evinces the view that the marmoset is secondarily lissencephalic.

The possibility that lissencephaly could also be a derived trait prompted us to inspect the rest of the mammalian tree for other possible reversals. The previously mentioned finding that bRG are present also in the mouse (albeit at low numbers) (Shitamukai et al., 2011; Wang et al., 2011) introduced the possibility that the widely used model organism, the mouse, might also be secondarily lissencephalic (Hevner and Haydar, 2012) (Figure 3A). In addition to having a small number of bRG, the rodent lineage has a fossil record of big-bodied animals (Rinderknecht and Blanco, 2008). The combination of the record of dwarfing in the rodent lineage, together with the fact that there are some members of the rodent order that are gyrencephalic (e.g., capybara), makes secondary lissencephaly a plausible explanation for brain morphology in the rodent order.

The most peculiar candidate for a secondarily lissencephalic species is the manatee (together with the whole order of Sirenia) (Figure 3A). Despite their large body size, the manatee brain is fairly lissencephalic, with only one deep gyrus (Reep and O'Shea, 1990). Although the fossil records indicate that even early sirenians were lissencephalic (O'Shea and Reep, 1990), their phylogenetic position among gyrencephalic lineages makes it possible that, due to the transition to aquatic environment, the whole lineage switched to a simplified gyrification pattern early on in evolution. The possible physiological reasons will be discussed later (see Adaptive Advantages of Lissencephaly Over Gyrencephaly).

The hypothesis of secondary lissencephaly is difficult to approach experimentally, as we lack the genetic determinants of the trait. Even the palaeontological record does not usually suffice to show it with high confidence because of the very low incidence of brain preservation, and especially because of the low frequency of the preservation of the gyrification pattern. Therefore, to show the plausibility of our hypothesis, we must revert to an *in silico* approach of modeling ancestral character traits. Indeed, the first models show that the secondary lissencephaly hypothesis is plausible (Kelava et al., 2012) and, as a matter of fact, a common process in the evolution of mammals (Lewitus et al., 2013). In general, the loss, or the variable expression of, a trait appearing relatively late in ontogeny is in accord with the notion that these stages of development are more amenable to evolutionary modification (Kalinka and Tomancak, 2012).

Nonetheless, an example of the process “in action” would provide even more support. The family of weasels (Mustelidae) might give us an insight into the process. The family has many species (Nowak, 1999), and has evolved quite rapidly after the divergence of small mammals (Koepfli et al., 2008). The fossil record shows that the ancestor of extant weasels (e.g., *Mustela putorius*, *Mustela erminea* and *Mustela nivalis*) was a larger animal (Kurtén, 2007). If we compare the brains of these three species (Figure 3B), we find that the smallest, the least weasel (*Mustela nivalis*), has a simpler pattern of gyrification than the bigger-bodied species. The gyri are also shallower. Therefore, rather than an abrupt loss of individual gyri, the process might have been gradual, with gyri becoming shallower as miniaturization progressed. This would be in accordance with the accepted theory of body and organism miniaturization which states that decreases in size lead to structural simplification (Hanken and Wake, 1993).

## Mechanisms of phenotypic reversal

The fact that many lineages may have transitioned from gyrencephaly to lissencephaly motivated workers to consider possible pathways by which different lineages would accomplish a similar phenotype. We considered the following possibilities: decrease of the number of bRG, downstream differences in the bRG lineage, changes in cell-biological parameters (e.g., duration of cell cycle), or changes in the timing of developmental events (heterochrony). Individual lineages might have opted for one or a combination of these pathways.

Recent advances in the field have established that the ancestor of Eutherian mammals was probably a gyrencephalic animal (O'Leary et al., 2013). The same seems to be true for the ancestor of all mammals (Lewitus et al., 2013). Therefore, all of the lissencephalic lineages present today would have to experience a loss of gyri (strictly speaking, all of them would be secondarily lissencephalic). How can this loss be established from a cell-biological point of view? One possibility is that the simpler, lissencephalic neocortex was accomplished by the loss of bRG, which in turn results in a smaller number of neurons. An example of this situation can be found in the mouse, in which a small population of bRG still persists (Shitamukai et al., 2011; Wang et al., 2011).

On the other hand, the marmoset's lissencephaly might have been accomplished by different means. The marmoset possesses a gyrencephaly-like cellular composition of the developing neocortex (García-Moreno et al., 2012; Kelava et al., 2012). By lengthening the cell-cycle, the marmoset might have produced a smaller number of neurons in order to give rise to a lissencephalic neocortex, while maintaining the necessary cytoachitecture to establish a gyrencephalic neocortex. By shortening the neurogenic window, the marmoset may have restricted the period in which neurons are born and thus decreased the final number of neurons. Similar cases of heterochrony, resulting in different sizes of brain parts, have been reported for birds (Charvet et al., 2011).

Changes in other cell-biological properties of neural progenitors are also possible. Our present knowledge of cell-biological properties of progenitor cells is most comprehensive for the mouse. This especially holds true for the proliferative and neurogenic potential of different progenitor populations. What is known is that bRG in different species have different proliferative potential and probably different daughter cell fate. The low numbers of bRG present in the mouse give rise to neurons through a self-consuming division (Shitamukai et al., 2011; Wang et al., 2011). The bRG in the human, on the other hand, can proliferate in the early stage of development and later give rise to transit-amplifying progenitor cells (TAPs), which enlarge the final neuronal pool immensely (Hansen et al., 2010). The fate of bRG in other described species (ferret and marmoset) is still uncertain, but it has been suggested that the bRG in the ferret produce, maybe in addition to a small number of TAPs, mostly neurons and, later, astrocytes (Reillo et al., 2011). The marmoset's bRG, on the other hand, might produce only neurons. These downstream differences might be another route for adjusting the final neuronal outcome and the gross morphology of the brain. Detecting these downstream differences in atypical model organisms is, in the absence of established techniques and a specific marker for bRG, still limited to static descriptive observations. The use of previously established markers Pax6 (Walther and Gruss, 1991; Götz et al., 1998; Osumi et al., 2008; Fietz et al., 2010; Hansen et al., 2010; Reillo et al., 2011) and Sox2 (Graham et al., 2003; Hansen et al., 2010) show that the bRG populations of the two analyzed species (ferret and marmoset) display some differences on the cell-biological level, which could be attributed to their differences in neuron-producing potential (Kelava, 2012).

These pathways might be a way to reconcile the relatively low number of different progenitor cell populations with the vast variability in the neuronal number and morphology present among mammalian species. They would also represent examples of convergent evolution.

## Adaptive advantages of lissencephaly over gyrencephaly

As we have seen from the examples mentioned above, brain morphology is a very plastic trait, which can undergo exhaustive changes to more complex or simpler forms during the evolution of a lineage. The fact that gyrencephaly is not dependent solely on phylogeny, and that we see similar patterns of brain morphology emerging in lineages which have undergone a process of miniaturization (e.g., marmosets, mice), or have inhabited a specific ecological niche (aquatic mammals), speaks in favor of it not being simply a consequence of physical properties of brain development, but being a selected trait. We can only speculate about the adaptive value of a folded or a smooth neocortex at the moment. Maybe, in small-bodied species, it is too energetically expensive to produce a folded neocortex. Also, smaller brains may not support highly folded neocortices simply for neuroanatomical reasons—the thickness of myelin sheets might interfere with proper patterns of connectivity in a small, gyrencephalic brain, rendering lissencephalic appearance in smaller brains adaptive. Therefore, in all species that underwent body size reduction, the simplification of gyrification pattern followed.

The fact that aquatic mammals exhibit peculiar patterns of gyrencephaly might also tell us something about environmental pressures (or constraints) imposed on neocortex morphology. Cetaceans (whales and dolphins) and pinnipeds (aquatic carnivores) have neocortices which are highly folded (Hadžiselimović and Dilberović, 1977; Butti et al., 2011; Manger et al., 2012). In whales this trait is so pronounced that their brains are described as *polymicrogyric* (Pilleri, 1964; Butti et al., 2011). In sirenians (manatees and dugongs) (Figure 3A), on the other hand, the brain is almost completely lissencephalic, despite their large body size (several hundred kilograms) (Reep et al., 1989). The fact that all groups of aquatic mammals have modified their gyrification pattern in some way, as compared to their closest relatives, could mean that the transition to water led to a relaxation of a terrestrial constraint. Whether this constraint is the gravitation pull which is diminished by living in water, we can only theorize. Some researchers propose that the constraints were not removed, but actually appeared with the terrestrial-to-aquatic transition. Manger (2006) stresses the need for better thermoregulation as a key feature that governed the evolution of the neocortices of aquatic mammals. In addition to this, water, as a medium so different to air in its physical properties, might have induced changed in the sensory cortex, leading to a modified neocortical morphology. In the case of sirenians, different postnatal growth rates for the brain and the body have been implicated in the development of the peculiarly lissencephalic brain and were maybe driven by similar constraints as in the cetaceans (Reep and O'Shea, 1990). The reason why the sirenian lineage took a completely different approach to cetaceans and pinnipeds maybe lies in their different habitat, feeding strategy and/or evolutionary rate. With our present knowledge, any discussion about the adaptive value of gyrencephaly is at best speculative. Nevertheless, novel insights into the extent of gyrencephaly across the mammalian clade speak to an intrinsic, cell-biological constraint, more than one involved purely with connectivity or spatial restriction.

**Figure 4:**
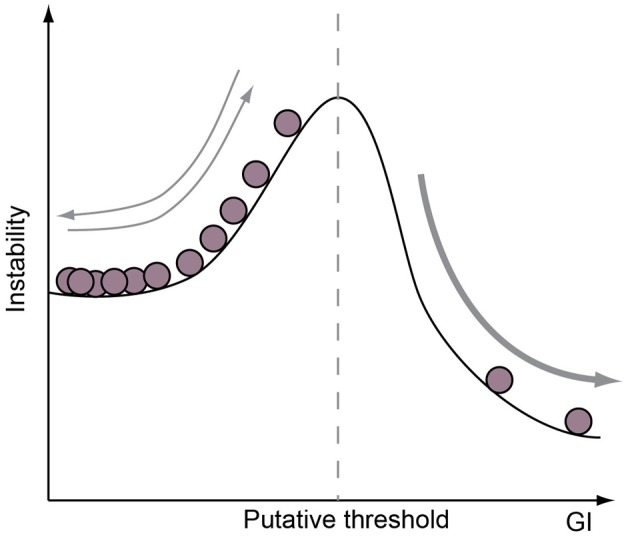
**Neurogenic program is a limiting factor on GI potential.** A cartoon illustrating the concept that the range of GI achievable is determined by the cell-biological features of a species neurogenic program. For example, species without proliferative progenitor-types in the basal compartment may be limited to GIs below a certain value (dotted vertical line), whereas species with such a progenitor-type are only constrained by a lower GI limit. Note that if a range of GI values may be achieved without adapting cell-biological features of the neurogenic period, then species in the lower GI range are likely to show frequent evolutionary increases and decreases in GI.

The adaptive value of brain size has often been described as a function of body size (Jerison, 1973; Shultz and Dunbar, 2010). This puts neocortical expansion on a continuous gradient, where competing selection pressures for brain and body size compete for an organism's metabolic potential. If that is the case, then the adaptive value of brain tissue per gram comes cheaper in smaller-brained species, where minor increases in brain size confers major alterations in brain-body ratios and, more specifically, investment in neocortical expansion must not only be two-fold more adaptive in tarsiers than bears, but shrews should be considered the summit of cerebral evolution in mammals (Roth and Dicke, 2005). We doubt anyone would argue either of these points. Rather, recent work has shown that both the total number of cortical neurons and the relative neuron density between cortical regions have order-specific scaling laws as functions of brain volume in primates, rodents, and carnivores, despite considerable deviations from brain-body scaling within each order (Herculano-Houzel, 2011; Lewitus et al., 2012). Furthermore, across all mammalian species, cortical surface area increases have a tendency to outpace evolutionary expansions in brain volume (Mayhew et al., 1996; Manger et al., 2012). If we couple these observations, then we may predict that, first of all, body size may be more constrained than brain size in mammals and, more pressingly, the adaptive value of neocortical expansion may be meaningfully measured by its number of folds. This latter prediction assumes—and correctly, as evidence in this review demonstrates—that adaptations to gross cortical morphology are underwritten by cell-biological variation across species. Specifically, and contrary to deviations from brain-body scaling relationships, there is a developmental correlate to cortical folding. The evidence for that developmental correlate, furthermore, shows that different neurodevelopmental paradigms (e.g., the relative abundance of bRG) may each produce a range of neocortical phenotypes, which may, in fact, explain the plasticity of gyrencephaly index [GI, a measure of the folding of the neocortex, Zilles et al. (1988)] at certain levels: if only minor perturbations in a neurodevelopmental program are necessary to generate a range of GI values, then we should expect gains and losses of global gyrencephaly to be manifested along myriad mammalian lineages. We should also expect that, once a cell-biological novelty is gained or lost along a lineage (e.g., neuron production from bRG via TAPs), then the range of GI values possible to species along that lineage will be significantly modified (Figure 4). Therefore, the adaptive window for secondary loss of gyrencephaly may be considerably constrained in species that are developmentally capable of achieving high levels of gyrencephaly and, correspondingly, the adaptive value of secondary lissencephaly may be tempered by the modest developmental cost of variation at low levels of gyrencephaly.

## Conclusion

**Figure 5:**
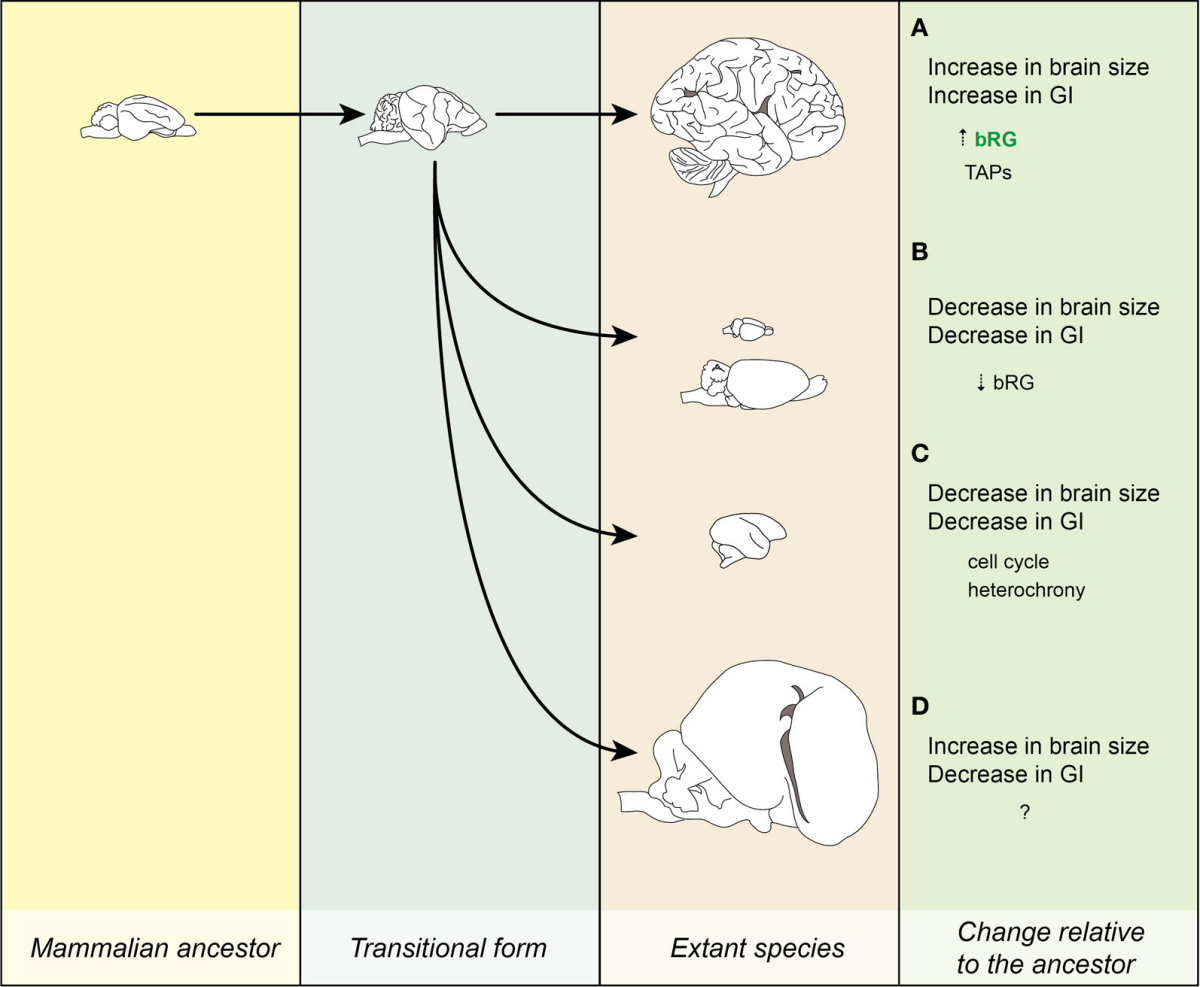
**Evolution of the neocortex.** Lineages leading to extant species. **(A)** Increase in brain size led to the increase in GI. The processes leading to this transition might have been that the bRG were present at a certain significant number (marked by green letters) and that they have evolved the ability to produce TAPs. Example: human. **(B)** Dwarfing I. Decrease in brain and body size relative to the ancestor is accompanied by a reduction in GI. The reduction in the number of bRG might have underlain this transition. Examples: mouse and beaver. **(C)** Dwarfing II. A similar process as in **(B)**, but the lissencephaly is due to changes in cell-biological parameters of progenitor cells (e.g., cell cycle), not their numbers. Example: marmoset. **(D)** Increase in brain and body size is accompanied by a decrease in GI. Example: manatee. Images for the ancestor and the transitional form are for illustration purposes only. Images drawn from photos obtained from www.brainmuseum.org and are not to scale.

Although the gross brain morphology has been studied for many years (Turner, 1890; Zilles et al., 2013), the underlying physical and cell-biological mechanisms have started to come to light only recently. The classical view of the evolution of the brain (ancestor with small/lissencephalic brain → species with big/gyrencephalic brain) gives way to a more complex concept of neocortical evolution (Figure 5). What has become evident is that brain morphology is a very plastic trait, which can be relatively easily altered in the course of evolution, by changing the ratios of different progenitor cells and some of their cell-biological features (e.g., cell cycle length). The extraordinary multiplication of neuron number along certain lineages may be due to the appearance of TAPs, a progenitor cell-type that may underwrite vast expansion of the neocortex observed along certain lineages. This more complex concept of neocortical evolution also brings ecological factors to light. The neocortex's need to adapt rapidly to ever changing environments underlies its flexibility, making the brain one of the more morphologically variable and plastic organs in the mammalian lineage.

Recent findings that the ancestor of mammals was probably a gyrencephalic animal (Lewitus et al., 2013; O'Leary et al., 2013) would imply that all lissencephalic lineages present today underwent a phenotypic reversal to a simpler form. For this reason, the focus of brain research which until now has mostly been centered on its evolutionary expansion (Kriegstein et al., 2006; Rakic et al., 2009), might have to shift also toward miniaturization. In other words, by studying the mouse, we are actually studying the secondary loss in the rodent lineage, instead of gain in the human lineage.

Phenotypic reversal (actually evolutionary reversal in general) is not an infrequent occurrence. Various examples of phenotypic and genomic reversions have been documented (Teotónio and Rose, 2001; Porter and Crandall, 2003) and one of the attributes of evolutionary reversal has been the ability to explore new evolutionary trajectories (Borowsky and Wilkens, 2002; Porter and Crandall, 2003). Maybe the ability of the brain to undergo quick phenotypic reversals is what is in the background of the immense adaptiveness of mammals.

The fact that gyrencephaly as a trait is genetically encoded (stability of gyrification pattern, gyrification pattern disorders) means that is can be targeted by natural selection and therefore is able to change in accordance to the needs of a particular population. It is therefore necessary to concentrate on elucidating this genetic make up of gyrification in order to study its adaptive value and its potential medical implications.

### Conflict of interest statement

The authors declare that the research was conducted in the absence of any commercial or financial relationships that could be construed as a potential conflict of interest.

## References

[B1] Barron D. H. (1950). An experimental analysis of some factors involved in the development of the fissure pattern of the cerebral cortex. J. Exp. Zool. 113, 553–581 10.1002/jez.1401130304

[B2] Borowsky R. Wilkens H. (2002). Mapping a cave fish genome: polygenic systems and regressive evolution. J. Hered. 93, 19–21 10.1093/jhered/93.1.19 12011170

[B3] Butti C. Raghanti M. A. Sherwood C. C. Hof P. R. (2011). The neocortex of cetaceans: cytoarchitecture and comparison with other aquatic and terrestrial species. Ann. N.Y. Acad. Sci. 1225, 47–58 10.1111/j.1749-6632.2011.05980.x 21534992

[B4] Charvet C. J. Striedter G. F. Finlay B. L. (2011). Evo-devo and brain scaling: candidate developmental mechanisms for variation and constancy in vertebrate brain evolution. Brain Behav. Evol. 78, 248–257 10.1159/000329851 21860220 PMC3221253

[B5] Cope E. (1896). The Primary Factors of Organic Evolution. Chicago: The Open Court Publishing Company

[B6] Fietz S. A. Kelava I. Vogt J. Wilsch-Bräuninger M. Stenzel D. Fish J. L. (2010). Osvz progenitors of human and ferret neocortex are epithelial-like and expand by integrin signaling. Nat. Neurosci. 13, 690–699 10.1038/nn.2553 20436478

[B7] Ford S. M. (1980). Callitrichids as phyletic dwarfs, and the place of the callitrichidae in platyrrhini. Primates 21, 31–34 10.1007/BF02383822

[B8] García-Moreno F. Vasistha N. A. Trevia N. Bourne J. A. Molnár Z. (2012). Compartmentalization of cerebral cortical germinal zones in a lissencephalic primate and gyrencephalic rodent. Cereb. Cortex 22, 482–492 10.1093/cercor/bhr312 22114081

[B9] Götz M. Huttner W. B. (2005). The cell biology of neurogenesis. Nat. Rev. Mol. Cell Biol. 6, 777–788 10.1038/nrm1739 16314867

[B10] Götz M. Stoykova A. Gruss P. (1998). Pax6 controls radial glia differentiation in the cerebral cortex. Neuron 21, 1031–1044 10.1016/S0896-6273(00)80621-2 9856459

[B11] Graham V. Khudyakov J. Ellis P. Pevny L. (2003). Sox2 functions to maintain neural progenitor identity. Neuron 39, 749–765 10.1016/S0896-6273(03)00497-5 12948443

[B12] Hadžiselimović H. Dilberović F. (1977). The appearance of the otter brain. Acta Anat. (Basel) 97, 387–392 10.1159/000144758 857567

[B13] Hanken J. Wake D. (1993). Miniaturization of body-size – organismal consequence and evolutionary significance. Ann. Rev. Ecol. Syst. 24, 501–519 10.1146/annurev.es.24.110193.002441

[B14] Hansen D. V. Lui J. H. Parker P. R. L. Kriegstein A. R. (2010). Neurogenic radial glia in the outer subventricular zone of human neocortex. Nature 464, 554–561 10.1038/nature08845 20154730

[B15] Haubensak W. Attardo A. Denk W. Huttner W. B. (2004). Neurons arise in the basal neuroepithelium of the early mammalian telencephalon: a major site of neurogenesis. Proc. Natl. Acad. Sci. USA 101, 3196–3201 10.1073/pnas.0308600100 14963232 PMC365766

[B16] Herculano-Houzel S. (2011). Not all brains are made the same: new views on brain scaling in evolution. Brain Behav. Evol. 78, 22–36 10.1159/000327318 21691045

[B17] Hevner R. F. Haydar T. F. (2012). The (not necessarily) convoluted role of basal radial glia in cortical neurogenesis. Cereb. Cortex 22, 465–468 10.1093/cercor/bhr336 22116731 PMC3256413

[B18] Jerison H. (1973). Evolution of the Brain and Intelligence. New York, NY: Academic Press

[B19] Kalinka A. T. Tomancak P. (2012). The evolution of early animal embryos: conservation or divergence? Trends Ecol. Evol. 27, 385–393 10.1016/j.tree.2012.03.007 22520868

[B20] Kelava I. (2012). Basal Radial Glia in Mammalian Neocortical Development – Insights Into Brain Evolution. Ph.D. thesis, Technische Universität Dresden.

[B21] Kelava I. Huttner W. B. (2012). Neurogenesis in the developing mammalian neocortex, in eLS. Chichester: John Wiley and Sons Ltd Available online at: http://www.els.net 10.1002/9780470015902.a0022541

[B22] Kelava I. Reillo I. Murayama A. Y. Kalinka A. T. Stenzel D. Tomancak P. (2012). Abundant occurrence of basal radial glia in the subventricular zone of embryonic neocortex of a lissencephalic primate, the common marmoset *Callithrix jacchus*. Cereb. Cortex 22, 469–481 10.1093/cercor/bhr301 22114084 PMC3256412

[B23] Koepfli K.-P. Deere K. A. Slater G. J. Begg C. Begg K. Grassman L. (2008). Multigene phylogeny of the mustelidae: resolving relationships, tempo and biogeographic history of a mammalian adaptive radiation. BMC Biol. 6:10 10.1186/1741-7007-6-10 18275614 PMC2276185

[B24] Kriegstein A. Noctor S. Martínez-Cerdeño V. (2006). Patterns of neural stem and progenitor cell division may underlie evolutionary cortical expansion. Nat. Rev. Neurosci. 7, 883–890 10.1038/nrn2008 17033683

[B25] Kurtén B. (2007). Pleistocene Mammals of Europe. New Brunswick, NJ: Aldine Transaction

[B26] Lewitus E. Kelava I. Kalinka A. T. Tomancak P. Huttner W. B. (2013). An Adaptive Threshold in Mammalian Neocortical Evolution. ArXiv e-prints: arXiv:1304.5412. 10.1371/journal.pbio.1002000 PMC4236020 25405475

[B27] Lewitus E. Sherwood C. C. Hof P. R. (2012). Cellular signatures in the primary visual cortex of phylogeny and placentation. Brain Struct. Funct. 217, 531–547 10.1007/s00429-011-0338-5 21863312

[B28] Lui J. H. Hansen D. V. Kriegstein A. R. (2011). Development and evolution of the human neocortex. Cell 146, 18–36 10.1016/j.cell.2011.06.030 21729779 PMC3610574

[B29] Luo Z.-X. (2007). Transformation and diversification in early mammal evolution. Nature 450, 1011–1019 10.1038/nature06277 18075580

[B30] Manger P. R. (2006). An examination of cetacean brain structure with a novel hypothesis correlating thermogenesis to the evolution of a big brain. Biol. Rev. Camb. Philos. Soc. 81, 293–338 10.1017/S1464793106007019 16573845

[B31] Manger P. R. Prowse M. Haagensen M. Hemingway J. (2012). Quantitative analysis of neocortical gyrencephaly in african elephants (loxodonta africana) and six species of cetaceans: comparison with other mammals. J. Comp. Neurol. 520, 2430–2439 22237903 10.1002/cne.23046

[B32] Martínez-Cerdeño V. Cunningham C. L. Camacho J. Antczak J. L. Prakash A. N. Cziep M. E. (2012). Comparative analysis of the subventricular zone in rat, ferret and macaque: evidence for an outer subventricular zone in rodents. PLoS ONE 7:e30178 10.1371/journal.pone.0030178 22272298 PMC3260244

[B33] Mayhew T. M. Mwamengele G. L. Dantzer V. Williams S. (1996). The gyrification of mammalian cerebral cortex: quantitative evidence of anisomorphic surface expansion during phylogenetic and ontogenetic development. J. Anat. 188(Pt 1), 53–58 8655415 PMC1167632

[B34] Miyata T. Kawaguchi A. Saito K. Kawano M. Muto T. Ogawa M. (2004). Asymmetric production of surface-dividing and non-surface-dividing cortical progenitor cells. Development 131, 3133–3145 10.1242/dev.01173 15175243

[B35] Montgomery S. H. Mundy N. I. (2013). Parallel episodes of phyletic dwarfism in callitrichid and cheirogaleid primates. J. Evol. Biol. 26, 810–819 10.1111/jeb.12097 23442013

[B36] Mota B. Herculano-Houzel S. (2012). How the cortex gets its folds: an inside-out, connectivity-driven model for the scaling of mammalian cortical folding. Front. Neuroanat. 6:3 10.3389/fnana.2012.00003 22347170 PMC3270328

[B37] Noctor S. C. Martínez-Cerdeño V. Ivic L. Kriegstein A. R. (2004). Cortical neurons arise in symmetric and asymmetric division zones and migrate through specific phases. Nat. Neurosci. 7, 136–144 10.1038/nn1172 14703572

[B38] Noctor S. C. Martínez-Cerdeño V. Kriegstein A. R. (2008). Distinct behaviors of neural stem and progenitor cells underlie cortical neurogenesis. J. Comp. Neurol. 508, 28–44 10.1002/cne.21669 18288691 PMC2635107

[B39] Nowak R. (1999). Walker's Primates of the World. Baltimore: Johns Hopkins University Press

[B40] O'Leary M. A. Bloch J. I. Flynn J. J. Gaudin T. J. Giallombardo A. Giannini N. P. (2013). The placental mammal ancestor and the post-k-pg radiation of placentals. Science 339, 662–667 10.1126/science.1229237 23393258

[B41] Olson E. C. Walsh C. A. (2002). Smooth, rough and upside-down neocortical development. Curr. Opin. Genet. Dev. 12, 320–327 10.1016/S0959-437X(02)00305-2 12076676

[B42] O'Shea T. J. Reep R. L. (1990). Encephalization quotients and life-history traits in the sirenia. J. Mammal. 71, 534–543 10.2307/1381792

[B43] Osumi N. Shinohara H. Numayama-Tsuruta K. Maekawa M. (2008). Concise review: Pax6 transcription factor contributes to both embryonic and adult neurogenesis as a multifunctional regulator. Stem Cells 26, 1663–1672 10.1634/stemcells.2007-0884 18467663

[B44] Pillay P. Manger P. R. (2007). Order-specific quantitative patterns of cortical gyrification. Eur. J. Neurosci. 25, 2705–2712 10.1111/j.1460-9568.2007.05524.x 17459107

[B45] Pilleri G. (1964). Morphologie des gehirnes des “southern right whale”, eubalaena australis desmoulins 1822 (cetacea, mysticeti, balaenidae). Acta Zool. 45, 245–272 10.1111/j.1463-6395.1964.tb00721.x

[B46] Porter M. Crandall K. (2003). Lost along the way: the significance of evolution in reverse. Trends Ecol. Evol. 18, 541–547 10.1016/S0169-5347(03)00244-1

[B47] Rakic P. (1988). Specification of cerebral cortical areas. Science 241, 170–176 10.1126/science.3291116 3291116

[B48] Rakic P. (2009). Evolution of the neocortex: a perspective from developmental biology. Nat. Rev. Neurosci. 10, 724–735 10.1038/nrn2719 19763105 PMC2913577

[B49] Rakic P. Ayoub A. E. Breunig J. J. Dominguez M. H. (2009). Decision by division: making cortical maps. Trends Neurosci. 32, 291–301 10.1016/j.tins.2009.01.007 19380167 PMC3601545

[B50] Reep R. L. Johnson J. I. Switzer R. C. Welker W. I. (1989). Manatee cerebral cortex: cytoarchitecture of the frontal region in trichechus manatus latirostris. Brain Behav. Evol. 34, 365–386 10.1159/000116523 2611642

[B51] Reep R. L. O'Shea T. J. (1990). Regional brain morphometry and lissencephaly in the sirenia. Brain Behav. Evol. 35, 185–194 10.1159/000115866 2379080

[B52] Reillo I. de Juan Romero C. García-Cabezas M. Á. Borrell V. (2011). A role for intermediate radial glia in the tangential expansion of the mammalian cerebral cortex. Cereb Cortex. 21, 1674–1694 10.1093/cercor/bhq238 21127018

[B53] Richman D. P. Stewart R. M. Hutchinson J. W. Caviness V. S. Jr. (1975). Mechanical model of brain convolutional development. Science 189, 18–21 10.1126/science.1135626 1135626

[B54] Rinderknecht A. Blanco R. E. (2008). The largest fossil rodent. Proc. Biol. Sci. 275, 923–928 10.1098/rspb.2007.1645 18198140 PMC2599941

[B55] Romiguier J. Ranwez V. Douzery E. J. P. Galtier N. (2013). Genomic evidence for large, long-lived ancestors to placental mammals. Mol. Biol. Evol. 30, 5–13 10.1093/molbev/mss211 22949523

[B56] Roth G. Dicke U. (2005). Evolution of the brain and intelligence. Trends Cogn. Sci. 9, 250–257 10.1016/j.tics.2005.03.005 15866152

[B57] Shitamukai A. Konno D. Matsuzaki F. (2011). Oblique radial glial divisions in the developing mouse neocortex induce self-renewing progenitors outside the germinal zone that resemble primate outer subventricular zone progenitors. J. Neurosci. 31, 3683–3695 10.1523/JNEUROSCI.4773-10.2011 21389223 PMC6622781

[B58] Shultz S. Dunbar R. (2010). Encephalization is not a universal macroevolutionary phenomenon in mammals but is associated with sociality. Proc. Natl. Acad. Sci. U.S.A. 107, 21582–21586 10.1073/pnas.1005246107 21098277 PMC3003036

[B59] Singer K. Luo R. Jeong S.-J. Piao X. (2013). Gpr56 and the developing cerebral cortex: cells, matrix, and neuronal migration. Mol. Neurobiol. 47, 186–196 10.1007/s12035-012-8343-0 23001883 PMC3538897

[B60] Smart I. H. McSherry G. M. (1986a). Gyrus formation in the cerebral cortex in the ferret. I. description of the external changes. J. Anat. 146, 141–152 3693054 PMC1166530

[B61] Smart I. H. McSherry G. M. (1986b). Gyrus formation in the cerebral cortex of the ferret. II. description of the internal histological changes. J. Anat. 147, 27–43 3693076 PMC1261544

[B62] Smart I. H. M. Dehay C. Giroud P. Berland M. Kennedy H. (2002). Unique morphological features of the proliferative zones and postmitotic compartments of the neural epithelium giving rise to striate and extrastriate cortex in the monkey. Cereb. Cortex 12, 37–53 10.1093/cercor/12.1.37 11734531 PMC1931430

[B63] Striedter G. (2005). Principles Of Brain Evolution. Sunderland, MA: Sinauer Associates

[B64] Teotónio H. Rose M. R. (2001). Perspective: reverse evolution. Evolution 55, 653–660 10.1111/j.0014-3820.2001.tb00800.x 11392382

[B65] Turner W. (1890). The convolutions of the brain: a study in comparative anatomy. J. Anat. Physiol. 25(Pt 1), 105–153 17231891 PMC1328113

[B66] Van Essen D. C. (1997). A tension-based theory of morphogenesis and compact wiring in the central nervous system. Nature 385, 313–318 10.1038/385313a0 9002514

[B67] Waddington C. H. (1961). Genetic assimilation. Adv. Genet. 10, 257–293 10.1016/S0065-2660(08)60119-4 14004267

[B68] Walther C. Gruss P. (1991). Pax-6, a murine paired box gene, is expressed in the developing cns. Development 113, 1435–1449 1687460 10.1242/dev.113.4.1435

[B69] Wang X. Tsai J.-W. LaMonica B. Kriegstein A. R. (2011). A new subtype of progenitor cell in the mouse embryonic neocortex. Nat. Neurosci. 14, 555–561 10.1038/nn.2807 21478886 PMC3083489

[B70] Welker W. (1990). Why does cerebral cortex fissure and fold? A review of determinants of gyri and sulci, Chapter 10, in Cerebral Cortex, Vol. 8B, eds Jones E. G. Peters A. (Springer), 3–136

[B71] Zilles K. Armstrong E. Schleicher A. Kretschmann H. J. (1988). The human pattern of gyrification in the cerebral cortex. Anat. Embryol. (Berl) 179, 173–179 10.1007/BF00304699 3232854

[B72] Zilles K. Palomero-Gallagher N. Amunts K. (2013). Development of cortical folding during evolution and ontogeny. Trends Neurosci. 36, 275–284 10.1016/j.tins.2013.01.006 23415112

